# Clinical and Biological Characteristics of Severe Malaria in Children under 5 Years Old in Benin

**DOI:** 10.1155/2023/5516408

**Published:** 2023-09-20

**Authors:** Xiao Ma, Xin Fan, Kora Chabi Youssaou, Junfei Zhang, Xingyi Wang, Guoqiang Zheng, Shuping Tian, Yujing Gao

**Affiliations:** ^1^Department of Emergency, General Hospital, Ningxia Medical University, Yinchuan, China; ^2^Department of Biochemistry and Molecular Biology, School of Basic Medical Sciences, Ningxia Medical University, Yinchuan, China; ^3^Department of Ophthalmology, General Hospital, Ningxia Medical University, Yinchuan, China; ^4^Department of Internal Medicine, Hospital of Zone of Natitingou (Women's and Children's Hospital), Natitingou, Atacora Province, Benin; ^5^General Practice, People's Hospital of Ningxia, Yinchuan, China; ^6^Department of Pediatric, General Hospital, Ningxia Medical University, Yinchuan, China

## Abstract

**Background:**

Malaria is a global public health concern, mainly occurring in sub-Saharan Africa. Children infected with malaria are more likely to develop severe disease, which can be fatal. During COVID-19 in 2020, diagnosing and treating malaria became difficult. We analyzed the clinical characteristics and laboratory indicators of children with severe malaria in Benin to provide important information for designing effective prevention and treatment strategies to manage pediatric cases.

**Methods:**

Clinical characteristics of pediatric patients with severe malaria admitted to two hospitals in Benin (Central Hospital of Lokossa and Regional Hospital of Natitingou, located ∼650 kilometers apart) were collected from January to December 2020. Patients were grouped according to age (group A: 4–12 months old, group B: 13–36 months old, and group C: 37–60 months old), and clinical and laboratory indicators were compared. The incidences of severe pediatric malaria in both hospitals in 2020 were calculated. Inclusion, exclusion, and blood transfusion criteria were identified.

**Results:**

We analyzed 236 pediatric cases. The main clinical symptoms among all patients were severe anemia, vomiting, prostration, poor appetite, dysphoria, and dyspnea. Over 50% of patients in group A experienced vomiting and severe anemia. Most patients in group B had severe anemia and prostration. Delirium affected significantly more patients in group C than in groups A and B. In group C, the hemoglobin and hematocrit levels were significantly higher (*p* < 0.05), and the leukocyte count was significantly lower (*p* < 0.01) than in groups A and B. Parasitemia was significantly higher in group C than in group A (*p* < 0.01). Twelve deaths occurred.

**Conclusions:**

Severe pediatric malaria is seasonal in Benin. The situation in children under 5 years old is poor. The main problems are severe disease conditions and high fatality rates. Effective approaches such as prevention and early and appropriate treatment are necessary to reduce the malaria burden in pediatric patients.

## 1. Introduction

Malaria is a major global public health concern [1]. According to the World Health Organization (WHO), between 2019 and 2020, the estimated malaria cases increased from 213 million to 241 million and deaths increased from 534,000 to 602,000 in the African region. This region accounted for approximately 95% of cases and 96% of deaths globally, and 80% of all deaths in this region were among children aged under 5 years. Most cases are due to *Plasmodium falciparum*. The percentage of total malaria deaths among children aged under 5 years decreased from 87% in 2000 to 76% in 2019, but increased slightly to 77% in 2020 [2]. In addition to mortality and morbidity, malaria causes huge economic losses [3]. A WHO report estimated that $3.5 billion USD was spent on malaria-related concerns in 2020 alone [2].

The five malarial *Plasmodium* species that affect humans are *Plasmodium falciparum*, *Plasmodium vivax*, *Plasmodium malariae*, *Plasmodium ovale*, and *Plasmodium knowlesi*. *P. falciparum* is the main causative agent of malaria in endemic areas. Severe malaria is primarily due to *P. falciparum* infections, followed by *P. vivax* and *P. knowlesi* infections [4, 5]. The different species show clear regional differences in their distributions, with 99.7% of *P. falciparum* present in Africa [6]. The latest data show that in Africa, one child dies from *P. falciparum* infection every 2 minutes [7].

Malaria is a major public health concern in Benin. In 2019, malaria cases constituted >38% of outpatients and >60% of inpatients nationwide. *P. falciparum* is the primary species; *P. vivax*, *P. malariae*, and *P. ovale* are less common. In 2019, the incidences of malaria, malaria-related mortality, severe malaria, and severe pediatric malaria were 21.2%, 1.3‰, 1.7%, and 4.34%, respectively.

In March 2020, the WHO issued guidelines for responding to malaria during the COVID-19 pandemic [8]. These guidelines formed part of the broader guidance on maintaining essential health services during the COVID-19 response [9]. The first cases of COVID-19 in Benin were reported in March 2020, just as the country was planning the long-lasting insecticidal net (LLIN) campaign. Following the WHO's recommendation to continue implementing malarial control interventions during the COVID-19 pandemic [8], the Ministry of Health was authorized to continue the LLIN campaign. Benin also successfully conducted indoor residual spraying during the COVID-19 pandemic, spraying 350,349 structures, and completed four rounds of seasonal malaria chemoprevention in four health zones. Finally, the country has worked to sustain case management of malaria during the COVID-19 pandemic. This included ensuring sufficient supplies of essential malaria commodities (e.g., diagnostics and treatment) at the health-facility level. All of these were achieved despite the difficult circumstances due to COVID-19 [9, 10].

In 2020, our team conducted medical surveys of two hospitals in Benin. We collected and analyzed clinical data on children under 5 years old with confirmed malaria who were admitted to these hospitals from January to December 2020. This study was conducted to provide basic clinical information and recommendations for treating severe pediatric malaria in Benin.

## 2. Methods

### 2.1. Study Site

**Figure 1:**
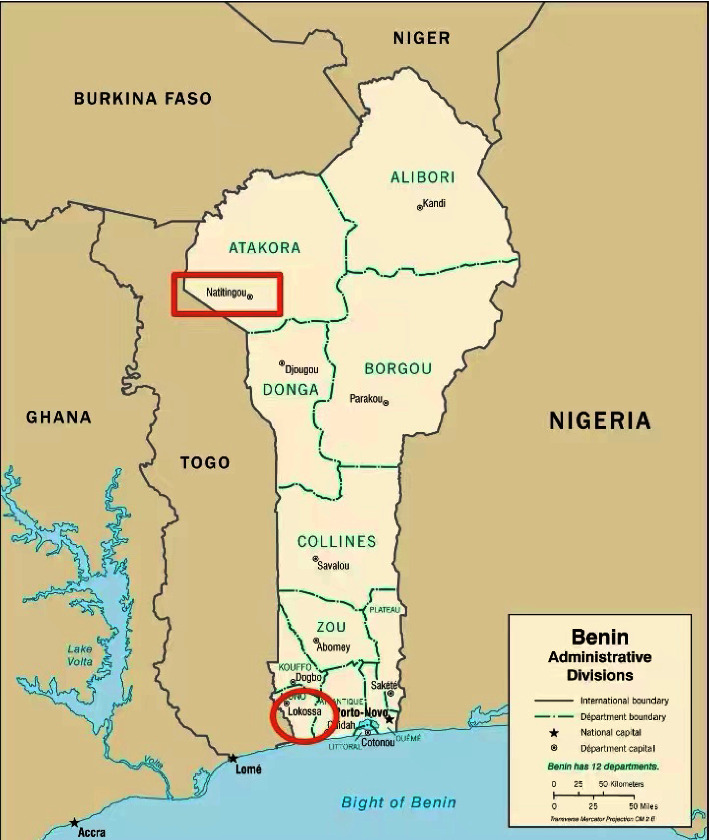
Locations of the two hospitals in Benin. The square indicates Natitingou; the circle indicates Lokossa. The map was obtained from https://mapsland.com/maps/africa/benin/large-detailed-administrative-divisions-map-of-benin-2007.jpg, with minor modifications.

The two hospitals in Benin involved in this study were the Central Hospital of Lokossa and the Regional Hospital of Natitingou. The Central Hospital of Lokossa is in northwest Mono-Couffo Province and is 106 km from Cotonou, the capital of the Republic of Benin. The Regional Hospital of Natitingou is in Accra Province, a hilly area in northern Benin. Both hospitals are located approximately 650 kilometers apart.  Figure 1 shows a map of Benin and the location of the two cities where these hospitals are located.

Benin, a narrow north-south key-shaped strip of land in West Africa, is situated between the Equator and the Tropic of Cancer. The country is divided into four main regions from south to north. The coastal plain region has a tropical rainforest climate, the central and northern regions have a savannah climate, and the south has a Guinea-type climate.

Lokossa in southwest Benin is a coastal area with elongated coastlines and extensive marshes. The city mainly consists of low-lying sandy coastal plains that stretch towards the Atlantic Ocean, along with marshes, lagoons, and lakes. Southern regions of Benin have two rainy periods from March to July and September to November, while the northern regions have a single rainy season from May to September. Natitingou, a town belonging to the Department of Atakora in northwestern Benin, lies in a semi-valley enclosed by two mountain ridges. The climate is drier in Natitingou than in the south, especially during the Harmattan season in December and January when humidity drops as low as 10% and night temperatures can reach as low as 17°C. Consequently, the climatic conditions in Lokossa are more favorable for mosquito breeding.

### 2.2. Case Collection/Data Collection

Clinical characteristics and indicators were recorded for the enrolled children with severe malaria, who were admitted to the two hospitals from 1 January 2020 to 31 December 2020. The patients were grouped according to age: group A (4–12 months old; 68 cases, 40 boys and 28 girls), group B (13–36 months old; 116 cases, 60 boys and 56 girls), and group C (37–60 months old; 52 cases, 32 boys and 20 girls). The patients' legal guardians provided informed written consent. The principles outlined in the Declaration of Helsinki were followed during the study.

Inclusion criteria were pediatric inpatients conformed to the diagnostic criteria for severe malaria, patients had consistent treatment plans, and cases included consent from the guardian and hospital director. Exclusion criteria were pediatric patients who did not conform to the diagnostic criteria for severe malaria, patients with different treatment options, patients whose guardians did not consent to their enrolling in the study, patients who quit before completing the treatment course, and patients with congenital diseases.

### 2.3. Diagnostic Criteria (Severe Malaria) [11–14]

The latest WHO guidelines for malaria treatment (third edition) and the Practical Manual for the Management of Severe Malaria (third edition) define severe malaria as follows: (1) positive microscopic examination or rapid diagnostic test for *P. falciparum* malaria with impaired consciousness (a Blantyre coma score of <3 of 5 in children); (2) prostration (inability to sit unassisted due to extreme weakness), multiple convulsions (generalized seizures), acidosis (plasma bicarbonate concentration <15 mM or intravenous plasma lactate level >5 mM), hypoglycemia (whole blood or plasma glucose concentration <2.2 mM or 40 mg/dL), severe anemia (hemoglobin <5 g/dL or hematocrit <15%), renal impairment (elevated plasma creatinine >265 *μ*M or blood urea >20 mM), jaundice (elevated plasma bilirubin >50 *μ*M with parasite count >100,000/*μ*L), pulmonary edema (tachypnea (respiratory rate >30/min)), dyspnea and hypoxia (oxygen saturation <92%), compensated shock (capillary refill ≥3 s without hypotension after rehydration), decompensated shock (systolic blood pressure <70 mmHg, with cool peripheries and prolonged capillary refill ≥3 s after adequate rehydration), or hyperparasitemia (parasitemia >10% without other severe signs).

### 2.4. Drug Treatments

Artemisinin was injected at 3 mg/kg, three times daily for patients weighing ≥20 kg and at 2.4 mg/kg, three times daily for patients weighing <20 kg. Oral artemether was used twice daily at 12 h intervals for 3 days. Patients weighing 5–14 kg received 120 mg artemether; those weighing 15–24 kg received 240 mg artemether; those weighing 25–34 kg received 360 mg artemether; and those weighing >34 kg received 480 mg artemether.

Notably, because of Benin's hygiene conditions and medical level, treatment programs for severe malaria are not fully implemented according to global treatment programs for severe malaria.

### 2.5. Blood Transfusion Criteria

Both hospitals adhered to the WHO's transfusion criteria [14]. In high-transmission settings, a hematocrit of ≤12% or a hemoglobin concentration of ≤4 g/dL is an indication for blood transfusion, regardless of the child's clinical condition. In children with less severe anemia (hematocrit: 13–18%; hemoglobin: 4–6 g/dL), transfusion was considered for those with respiratory distress (acidosis), impaired consciousness, hyperparasitemia (>20%), shock, or heart failure. Hemoglobin (hematocrit) levels were rechecked after blood transfusions to determine whether further transfusion was required.

### 2.6. Evaluation of Treatment Effects

Cured was defined as the absence of clinical symptoms and no *Plasmodium* in peripheral blood films [15]. The major indicators of a poor prognosis in children with severe *P. falciparum*-associated malaria [14] used by the two hospital are as follows. Clinical indicators are age <3 years, deep coma, clinical signs of organ dysfunction (e.g., renal injury and pulmonary edema), respiratory distress (acidosis), and shock. Laboratory indicators are hyperparasitemia (>250,000/*µ*L or >5%), erythrocyte volume fraction <15%, hemoglobin concentration <5 g/dL, blood glucose <2.2 mmol/L (<40 mg/dL), blood urea >60 mg/dL, and serum creatinine >265 *µ*mol/L (>3.0 mg/dL).

### 2.7. Laboratory Indicators

*P. falciparum* was detected in the thick film of patients' peripheral blood under light microscopy after fixation and staining with Giemsa. The parasite load was calculated using the formula: number of parasites/number of white blood cells × exact white blood cell count. If the exact count was unknown, the average normal white blood cell count in Benin (6000 GB/UL) was used for the calculation [16]. The following indicators were recorded: hemoglobin level (reference interval: 115.0–150.0 g/L), hematocrit level (reference interval: 35.00%–45.00%), white blood cell count (reference interval: 3.50–9.50 × 10^9^/L), red blood cell count (reference interval: 3.80–5.10 × 10^12^/L), platelet count (reference interval: 125.0–350.0 × 10^9^/L), C-reactive protein level (normal reference value: <10.0 mg/L), and *Plasmodium* count (normal reference value: 0).

### 2.8. Statistical Analysis

Clinical characteristics and indicators were compared among groups. Statistical analysis was performed using SPSS software (version 20.0; SPSS, Chicago, IL, USA). Normally distributed data are expressed as means ± standard deviation and compared via *t*-tests. Nonnormally distributed data are expressed as medians with interquartile range and compared using nonparametric tests. Chi-square tests were used to compare categorical data. The statistical significance threshold was set at *p* < 0.05.

## 3. Results

### 3.1. Demographics

In total, 236 pediatric patients (132 boys, 104 girls, 1.27 : 1 ratio) with severe *P. falciparum*-induced malaria were analyzed. Twelve deaths occurred, yielding a fatality rate of 5.10%. We divided the 236 patients into groups A, B, and C according to age, as described in the Methods section, and compared the clinical characteristics and indicators among them (Table 1).

### 3.2. Incidence of Severe Pediatric Malaria in Benin

Benin has only rainy and dry seasons. Data from the two hospitals show that in 2020, the incidence of severe malaria in children was higher during the rainy seasons, especially from June to November, with the highest incidence in September (Table 2).

### 3.3. Clinical Symptoms

The main clinical symptoms among all patients were severe anemia, vomiting, prostration, poor appetite, dysphoria, and dyspnea. Some clinical symptoms differed among groups. More than 50% of patients in group A experienced vomiting and severe anemia, while most of group B had severe anemia and prostration. Delirium affected significantly more patients in group C than in groups A and B (Table 3).

### 3.4. Laboratory Indicators and Hospital Stay Lengths

In group C, the hemoglobin levels, hematocrit levels, and red blood cell counts were significantly higher (all *p* < 0.05), and the leukocyte count was significantly lower (*p* < 0.01) than in groups A and B (Table 4). The *Plasmodium* count was significantly higher in group C than in group A (*p* < 0.01). The remaining laboratory indicators and the average hospital stay lengths did not significantly differ among the groups (hospital stays: A: 5.07 ± 6.31 days vs. B: 5.15 ± 3.45 days vs. C: 5.67 ± 4.14 days; *p* > 0.05).

## 4. Discussion

In this study, most children had malignant malaria, characterized by severe anemia, vomiting, prostration, poor appetite, delirium, and dyspnea. Groups A, B, and C had distinct characteristics, indicating that symptoms differed by age. Moreover, severe pediatric malaria occurred more often in the rainy season, with September having the highest incidence. Malaria burden is strongly associated with environmental, climatic, seasonal and behavioral factors [17, 18]. Seasonal rainfall and temperature variability affect the breeding habitats and larval development of mosquito vectors and the growth rate of the malarial parasites within them [19, 20]. Benin is near the Atlantic Ocean, and its humidity is approximately 80%; the annual rainy season lasts 3-4 months, during which the number of mosquitoes increases. The two hospitals included in this study are in different geographic areas (one inland and one coastland); however, both showed similar climate characteristics. The relatively low temperature range, high relative humidity, and presence of permanent and semipermanent water bodies make these areas more suitable for mosquito breeding during rainy seasons.

Malaria is transmitted via bites from *Anopheles* mosquitoes, which exhibit biting activity between sunset and sunrise [21]. Over 400 species of *Anopheles* mosquitoes exist worldwide, of which approximately 30 are major vectors of malaria. The *Anopheles gambiae* complex, which spreads malaria in Africa, consists of eight cryptic species. *A. gambiae s.s.* and *A. coluzzii* are considered the dominant vector species [22]. In Benin, *A. melas* also transmits malaria [23]. The high-risk groups for malaria infection and for developing severe disease include children under 5 years old, pregnant women, individuals with human immunodeficiency virus, nonimmune immigrants, migrant populations, and travelers [10, 24].

Babies are believed to have partial protection from malaria in the first few months of life, owing to passively acquired maternal IgG antibodies, the predominance of hemoglobin F (HbF) in their erythrocytes, and the low levels of iron and para-amino benzoic acid in breast milk [25, 26]. Malaria infection in the first week of life is referred to as “congenital malaria,” while that occurring within the next 3 weeks is referred to as “neonatal malaria.” The former is assumed to be acquired from the mother, the latter by mosquito inoculation [11]. In this study, we selected children between 4 months and 5 years of age. Susceptibility to malaria is higher in these children because they have not yet developed adaptive immunity; therefore, *Plasmodium* develops more easily in these children, and *P. falciparum* can cause severe malaria.

Severe anemia is the leading cause of death in children with malaria and is a common presenting feature of *P. falciparum*-induced malaria in areas of high transmission. Severe anemia develops rapidly after infections with high parasitic density and occurs earlier and more significantly. In these cases, acute destruction of parasitized red blood cells, which causes the anemia, requires careful monitoring during treatment [14]. In our patients, the red blood cell counts, hemoglobin levels, and platelet counts were reduced to varying degrees, indicating that malaria affects nearly all blood components. Decreased platelet counts and anemia were the most common malaria-related hematological complications. All 236 patients had varying degrees of reduction in hemoglobin levels, 105 had severe anemia, and 192 required transfusions. Among peripheral blood laboratory indicators, the hemoglobin and hematocrit levels were significantly increased in group C compared with those in groups A and B, indicating that anemia was severer in groups A and B. The red blood cell counts in group C did not statistically differ from those in group A but were statistically increased compared with those of group B, indicating that anemia was severer in group B. Our results are consistent with those of other epidemiological studies that suggested that older children with malaria generally exhibit mild clinical symptoms, while younger children have severe clinical symptoms; thus, immune system development may contribute to the presentation of pediatric malaria [27, 28].

Platelet counts are a critical factor in diagnosing malaria [29]. Platelet counts were substantially decreased to 13 × 10^9^/L in some of our patients, which is consistent with previous findings [30]. Some possible reasons for this include the large numbers of phagocytosed *Plasmodium*-containing erythrocytes in the spleen, involvement of platelets in repairing *Plasmodium*-damaged vascular endothelial function, excessive platelet aggregation and damage caused by *Plasmodium* antigen-mediated immune mechanisms, and disrupted maturation of megakaryocytes in the bone marrow [31]. Therefore, malaria severity can be evaluated clinically according to whether platelet counts are decreased (<50 × 10^9^/L) [32, 33]. After *Plasmodium* removal, platelet counts can return to normal. In this study, platelet counts did not significantly differ among groups, indicating that platelet counts do not substantially change with age.

Leukocyte and neutrophil counts can increase during acute paroxysm, remain normal after the paroxysm, and decrease after multiple paroxysms [12]. In this study, leukocyte counts differed significantly among groups, presumably in relation to the immune system or number of paroxysms. Because the clinical data in this study are limited, further validation is needed.

Several factors might contribute to changes in C-reactive protein levels in patients with malaria. First, after *Plasmodium* infection, red blood cells experience endovascular adhesion damage. Second, *Plasmodium* parasites cause microvascular lesions and pathological reactions in the brain, lungs, and other organs. Third, *Plasmodium* parasites cause widespread secretion of C-reactive protein into blood [34]. In this study, C-reactive protein levels did not differ significantly among groups, indicating that these levels do not substantially change with age.

This study had some limitations. First, because of financial limitations, patients were generally hospitalized for 2–4 days. After patients received blood transfusions and antimalarial treatment, their guardians often requested discharge if the patient's condition was improved. If financial conditions allowed, the children were discharged with instructions to continue oral medication at home. If the financial conditions were poor, the children were only observed after returning home. Incomplete laboratory registration books were also a limitation.

In West African nations such as Benin, where the malaria incidence and mortality are high, comprehensive treatment programs are needed. Currently, West African nations with high malaria incidences have weak monitoring systems. Malaria must be eliminated as well as diagnosed and treated. The following factors should be considered: increasing investment in domestic and international resources to enable assessing malaria patients with uniform criteria; investing public health resources where the population has the greatest need and the intervention will be most effective; strengthening the training and technical guidance for local healthcare staff and developing appropriate local malaria elimination measures consistent with scientific assessment of the epidemic scope and transmission characteristics; and providing practical control packages (e.g., malaria control knowledge manual, mosquito nets, thermometer, mosquito repellent, and preventive drugs).

## 5. Conclusions

Severe malaria remains problematic in children under 5 years old. The main problems are disease conditions and high fatality rates. Effective approaches such as infection prevention and early and appropriate treatment are necessary to reduce the malarial burden in endemic settings. The international community should continue to pay attention to this life-threatening disease and assist special populations, such as young children and pregnant women, in diagnosing and treating malaria in sub-Saharan Africa.

## Figures and Tables

**Table 1:** Demographic characteristics of patients with severe pediatric malaria.

Group	*n*	Sex		Age (months) M (P_25_–P_75_)	Weight (kg) M (P_25_–P_75_)	Blood transfusion	
		Male	Female			Yes, *n* (%)	No, *n*
Group A	68	40	28	9 (7–12)	8 (7–9)	53 (77.94%)	15
Group B	116	60	56	24 (24–36)	11 (10–12)	100 (86.20%)	16
Group C	52	32	20	60 (48–60)	15 (14–18)	39 (75.00%)	13

*Statistics*							
*χ* ^2^/H	—	1.727		201.824	160.251	3.707	
*p* value	—	0.422		<0.001	<0.001	0.157	

*Notes*. M, median; P_25_–P_75_, first to third quartiles.

**Table 2:** Incidence of severe pediatric malaria in Natitingou and Lokossa hospitals from January to December (2020).

Month	Natitingou (%)	Lokossa (%)
January	44.49	54.18
February	52.31	50.56
March	75.98	50.28
April	46.77	53.62
May	47.39	65.04
June	90.77	87.80
July	92.93	87.55
August	90.28	90.86
September	92.85	93.65
October	86.99	84.21
November	89.40	87.24
December	74.35	75.02

*Note.* The percentage was the ratio of the number of severe pediatric malaria patients to the number of all pediatric malaria patients in each hospital (data sourced from the hospitals).

**Table 3:** Clinical symptoms of patients with severe pediatric malaria.

Clinical symptom	Group A	Group B	Group C	*χ* ^2^	*p* value
	*n* (%)	*n* (%)	*n* (%)		
Vomiting	37 (54.41)	45 (38.79)	20 (38.46)	4.877	0.087
Severe anemia	35 (51.47)	53 (45.69)	17 (32.69)	4.340	0.114
Prostration	29 (42.65)	54 (46.55)	25 (48.08)	0.407	0.816
Poor appetite	25 (36.76)	50 (43.10)	17 (32.69)	1.834	0.400
Dyspnea	21 (30.88)	38 (32.76)	11 (21.15)	2.386	0.303
Diarrhea	12 (17.65)	15 (12.93)	10 (19.23)	1.358	0.507
Coma	8 (11.76)	13 (11.21)	3 (5.77)	1.428	0.490
Renal impairment	6 (8.82)	29 (25.00)	17 (32.69)	10.941	0.004
Convulsions	5 (7.35)	11 (9.48)	4 (7.69)	0.014	0.907
Hematuria	2 (2.94)	17 (14.66)	9 (17.31)	7.515	0.023
Jaundice	2 (2.94)	7 (6.03)	3 (5.77)	0.571	0.450
Delirium	0 (0)	0 (0)	23 (44.23)	—	—

*Note.* Delirium was not statistically analyzed because groups A and B had no cases; thus, the standard deviation was excessive.

**Table 4:** Laboratory indicators among groups of patients with severe pediatric malaria.

Laboratory indicators	Group A	Group B	Group C	*p* value	
				A vs. C	B vs. C
HGB (g/L)	53.63 ± 23.48	52.32 ± 20.48	61.96 ± 21.49	0.020	0.005
HCT (%)	17.18 ± 7.35	16.52 ± 6.48	20.52 ± 10.02	0.020	0.002
RBC (^*∗*^10^12^/L)	238.14 ± 95.66	236.64 ± 88.08	272.13 ± 103.03	0.050	0.020
*Plasmodium* (Pu/L ^*∗*^10^3^)	105.47 ± 232.7	712.01 ± 1375.68	974.99 ± 1988.37	0.001	0.200
WBC (^*∗*^10^9^/L)	13.61 ± 9.51	13.89 ± 11.33	9.54 ± 5.72	0.005	0.005
PLT (^*∗*^10^9^/L)	118.20 ± 98.38	106.46 ± 92.91	90.66 ± 81.22	0.100	0.200
CRP (mg/L)	73.95 ± 41.75	89.50 ± 57.22	74.43 ± 38.33	0.500	0.050

HGB, hemoglobin; HCT, hematocrit; RBC, red blood cells; WBC, white blood cells; PLT, platelets; CRP, C-reactive protein.

## Data Availability

The datasets used and/or analyzed during the current study are available from the corresponding authors upon request.

## Abbreviations

WHO: World Health Organization.
